# Impact of Molar Composition on the Functional Properties of Glutinous Rice Starch–Chitosan Blend: Natural-Based Active Coating for Extending Mango Shelf Life

**DOI:** 10.3390/polym16101375

**Published:** 2024-05-11

**Authors:** Chawakwan Nitikornwarakul, Rodjanawan Wangpradid, Natthida Rakkapao

**Affiliations:** 1 Faculty of Innovative Agriculture and Fishery Establishment Project, Prince of Songkla University, Surat Thani Campus, Surat Thani 84000, Thailand; chawakwan.n@ku.th (C.N.); rodjanawannn@gmail.com (R.W.); 2 Department of Food Science and Technology, Faculty of Agro-Industry, Kasetsart University, Bangkok 10900, Thailand; 3 Department of Applied Chemistry, Faculty of Science and Industrial Technology, Prince of Songkla University, Surat Thani Campus, Surat Thani 84000, Thailand; 4 Membrane Science and Technology Research Center, Faculty of Science, Prince of Songkla University, Hat Yai Campus, Songkhla 90110, Thailand

**Keywords:** starch, chitosan, blend composition, mango, green active coating, functional biopolymer, post-harvest preservation, anthracnose inhibition, antifungal, shelf-life extension

## Abstract

This study investigates natural-based blends of glutinous rice starch (GRS) and chitosan (CS), varying their molar composition (0:100, 30:70, 50:50, 70:30, and 100:0) to explore their interaction dynamics. Our findings illustrate the versatility of these blends in solution and film forms, offering applications across diverse fields. Our objective is to understand their impact on coatings designed to extend the post-harvest shelf life of mangoes. Results reveal that increasing chitosan content in GRS/CS blends enhances mechanical strength, hydrophobicity, and resistance to *Colletotrichum gloeosporioides* infection, a common cause of mango anthracnose. These properties overcome limitations of GRS films. Advanced techniques, including FTIR analysis and stereo imaging, confirmed robust interaction between GRS/CS blend films and mango cuticles, improving coverage with higher chitosan content. This comprehensive coverage reduces mango dehydration and respiration, thereby preserving quality and extending shelf life. Coating with a GRS/CS blend containing at least 50% chitosan effectively prevents disease progression and maintains quality over a 10-day storage period, while uncoated mangoes fail to meet quality standards within 2 days. Moreover, increasing the starch proportion in GRS/CS blends enhances film density, optical properties, and reduces reliance on acidic solvents, thereby minimizing undesirable changes in product aroma and taste.

## 1. Introduction

Fruits and vegetables serve as vital repositories of valuable nutrients, yet they continue to respire and transpire post-harvest, rendering them highly perishable. Horticultural crops worldwide experience significant post-harvest losses, estimated at around 1.3 billion tons annually, with developing countries facing more challenges [1]. In response to the imperative need for sustainable solutions in the food industry, natural-based active films have emerged as a promising alternative for extending the shelf life of fresh produce.

Comprised of biodegradable polymers and food-grade additives, these films are applied as thin layers in the form of wraps or coatings on food surfaces. The expanding environmental consciousness, coupled with regulatory restrictions on the use of agrochemicals and an escalating consumer demand for safe foods, has spurred a robust exploration of biodegradable films in post-harvest applications. Leveraging various biomaterials, including polysaccharides (such as starch, cellulose, chitosan, pectin, and alginate), proteins (including whey protein, casein, gelatin, collagen, soy protein, wheat gluten, and corn zein), and lipids (comprising natural waxes, acetylated monoglycerides, and resins) [2], these innovative films exhibit barrier properties that regulate water and gas exchange, thereby mitigating post-harvest losses.

Mango (*Mangifera indica* L.) stands as the second most economically significant tropical fruit commodity worldwide, with a global annual production reaching nearly 59 million tons in 2022 [3]. The fruit is renowned for its exceptional taste, aroma, and rich nutritional content. Classified as climacteric fruits, mangoes undergo a dramatic increase in respiration and ethylene production during ripening [4]. Upon harvesting from the tree, mangoes ripen quickly within 3–9 days at ambient temperatures [5]. During the onset of ripening, certain physiochemical changes take place, leading to the softening of the fruit tissue, thus increasing its vulnerability to microbial invasion [6,7]. Furthermore, mangoes are susceptible to various challenges, including water loss; microbial decay such as anthracnose and stem end rot; chilling injury; and mechanical damage, all of which hinder their handling and transportation [5]. Considering these challenges, preserving mango quality presents a significant challenge. Various approaches have been employed to maintain the quality of exported mangoes, including heat treatment, cold storage management, controlled atmosphere storage, 1-methylcyclopropene, ethylene, methyl jasmonate, and edible coatings [8]. Recent research has focused on utilizing natural materials as fruit coatings to reduce post-harvest diseases, preserve quality, and extend the shelf life of mangoes.

Starch stands out for its versatility, abundance, cost-effectiveness, edibility, and renewability, making it a prime choice for biodegradable film. Its unique characteristic and gelatinization properties have led to extensive utilization in this field [9]. Starch-based films have found success in delaying ripening and reducing weight/firmness loss in various fruits, such as avocados [10], pears [11], grapes [12], and cherry tomatoes [13]. Improving the barrier properties of starch-based films has been suggested by elevating starch crystallinity or amylopectin content. Furthermore, starch films with high amylopectin exhibit enhanced tensile strength, reduced moisture sensitivity, and increased crystallinity [14]. Glutinous rice starch (GRS), with its high amylopectin content (98–99%), exhibits exceptional gas barrier properties, rendering it an optimal candidate for investigation in this study. However, GRS alone may lack essential physicochemical, mechanical, and antimicrobial properties required for industrial applications. Thus, blending it with other biopolymers becomes imperative to enhance its properties, like water vapor and gas barrier abilities, along with antimicrobial activity.

Chitosan (poly-ß-(1,4)-D-glucosamine) has emerged as a prominent candidate in post-harvest applications, owing to its film-forming properties, biocompatibility, biodegradability, oxygen barrier ability, mechanical strength, and remarkable antimicrobial properties, particularly its anti-fungal ability [15]. The presence of reactive groups in chitosan, such as -OH and -NH_2_, plays a crucial role in its antimicrobial properties. Yan et al. [16] elucidated that the -NH_3_^+^ group of deacetylated chitosan interacts electrostatically with the phosphoryl groups of microbial cell membranes, resulting in cell leakage. Chitosan also exhibits anti-fungal properties by directly impeding fungal growth and activating specific biological processes in plant tissue [17]. Its ability to inhibit gas exchange between fruits and their surroundings, thereby reducing respiration rates, positions chitosan as a compelling option for extending the post-harvest longevity of diverse produce, such as berry fruit [18], fresh fig [19], and mango [20].

Combining starch and chitosan presents a compelling strategy to enhance the inherent properties of both polymers. Starch, though versatile, often falls short in mechanical strength, water resistance, and antimicrobial efficacy. Conversely, chitosan, prized for its antimicrobial ability, can introduce undesirable traits such as a yellowish hue and subtle alterations in taste and odor due to the use of acidic solvents. These factors directly influence consumer safety and acceptance. By synergizing these polymers, we aim to overcome these limitations and develop environmentally friendly, edible coatings or packaging solutions. Our research explores this innovative approach to extend the shelf life of produce and food, ensuring both safety and sustainability.

The utilization of composite starch/chitosan films has proven effective in enhancing the shelf life of fresh produce. Numerous studies have illustrated the beneficial impact of these films on extending shelf life. For instance, a cassava starch–chitosan coating mitigated weight loss and microbial spoilage in black mulberries stored at 5 °C for 16 days [21]. Starch films incorporating chitosan nanoparticles efficiently inhibited microbial growth in cherry tomatoes [22]. Furthermore, the application of a purple yam starch/chitosan film on apples for 4 weeks preserved fruit quality by reducing weight loss [23]. This study introduces a novel approach by proposing a coating composed of GRS and chitosan, with the aim of extending the shelf life of mangoes by mitigating anthracnose infection. Our primary objective is to explore the mechanical and functional characteristics of the GRS-CS film across various molar ratios, focusing on its potential as a biodegradable coating tailored specifically for mango preservation. Given the significant economic importance of Nam Dok Mai mangoes in Thailand and their widespread global distribution, this research offers a promising technology to enhance their shelf life in export markets. By addressing critical challenges related to mango preservation and transportation, our findings contribute to the advancement of post-harvest preservation strategies for this valuable commodity.

## 2. Materials and Methods

### 2.1. Materials

Glutinous rice starch (GRS) was sourced from Thai Flour Industry Co., Ltd. (Bangkok, Thailand). GRS has a repeat unit molecular weight of 162 Da. Chitosan flakes prepared from squid pens were purchased from Bio21 Co., Ltd. (Chonburi, Thailand). The chitosan used in this study possesses a high molecular weight exceeding 1000 kDa, along with a 95% degree of deacetylation, and the molecular weight of a repeat unit of chitosan is 161 Da. Mature mango fruit, which were harvested at 95–110 days old (*Mangifera indica* L. cv. Nam Dok Mai), were purchased from a commercial orchard in Ratchaburi, Thailand. The selected mangoes were uniform in size, shape, color, ripeness and lack of blemishes, injuries, and disease symptoms.

### 2.2. Methods

The study was structured into two main experimental phases: (I) exploring the impact of diverse molar proportions of starch to chitosan on GRS/CS solution and film properties; and (II) implementing GRS/CS active coatings with varying molar ratios on mango surfaces. This investigation aims to assess the feasibility of employing GRS/CS blends as active coatings to preserve the quality of post-harvest agricultural products, particularly fruits and vegetables.

#### 2.2.1. Experiment Part I: Assessing the Effects of Varying Molar Proportions of GRS to Chitosan on GRS/CS Solution and Film Properties

The GRS (2% *w*/*v*) and chitosan (2% *w*/*v*) solutions were prepared separately. GRS was dissolved in deionized water and stirred at 75–80 °C for 1 h, while chitosan solution was prepared in 1 M acetic acid and stirred at room temperature (~27 °C) for 48 h. The GRS and chitosan solutions were then mixed in different molar ratios (100:0, 70:30, 50:50, 30:70, 0:100) to yield a total volume of 100 mL, as outlined in Table 1, resulting in the formulation of coating solutions named GRS100/CS0, GRS70/CS30, GRS50/CS50, GRS30/CS70, and GRS0/CS100. The mixed polymer solutions were stirred at room temperature (~27 °C) for 30 min until clear and homogeneous solutions were obtained, and the pH of the solutions was recorded. To form films, the coating solution (30 mL) was carefully poured onto a 9 cm diameter plastic *Petri* dish and dried in a hot air oven at 50–55 °C for 24 h. The dried films were peeled off, placed in plastic Ziplock bag, and stored at 53 ± 1% RH and 27 ± 1 °C in desiccators prior to any testing.

##### Properties of GRS/CS Blended Solution

*Viscosity*

The viscosity of the coating solutions was measured using a Brookfield viscometer, Model LVD V3T (Ametex Brookfield, Middleborough, MA, USA). A 200 mL sample of each coating solution was placed in the viscometer and measured using spindle LV-2 at a speed of 40 rpm for 3 min at 27 °C. The viscosity of the coating solutions was obtained as the average of 3 replications.

*Effect of coating solution on Colletotrichum gloeosporioides mycelial growth*

The fungal strain *C. gloeosporioides* was obtained from the Plant Protection Research and Development office, Department of Agriculture, Bangkok, Thailand. A pure culture of *C. gloeosporioides* was sub-cultured on potato dextrose agar (PDA, Difco^TM^, Franklin Lakes, NJ, USA) in *Petri* dishes (9 cm diameter) and incubated at room temperature for 14 days until mycelial growth reached the dish’s margin.

The in vitro antifungal activity of the coating solution was evaluated using PDA amended with different concentrations of the coating solution. A 7 mm diameter agar plug from the margin of a 14-day-old pure culture of *C. gloeosporioides* was transferred to the center of each PDA plate supplemented with the desired concentration of GRS/CS coating solution (100:0, 70:30, 50:50, 30:70, 0:100 (% mol)). PDA plates containing 0.1% *v/v* aqueous acetic acid solution (pH 5.6) served as the negative control. Three replicates of each coating solution were prepared. All plates were then incubated at room temperature for 14 days, and the radial mycelial growth was measured daily in two perpendicular directions. The results are expressed as the percentage of mycelial growth inhibition (MGI), calculated using Equation (1), where D_c_ represents the colony diameter in the control plate and D_s_ represents the colony diameter in the PDA supplemented with a coating solution.

MGI (%) = [(D_c_ − D_s_)/D_c_] × 100 (1)

##### Morphology, Physical, and Mechanical Properties of GRS/CS Blend Films

*Morphology*

Scanning electron microscopy (SEM; Quanta400, FEI, Brno-Černovice, Czech Republic) was used to study variations in film morphology resulting from different GRS and chitosan blend compositions. Sample films were mounted with conductive adhesive tape, sputter-coated with gold, and observed at an accelerating voltage of 15 kV. The analysis encompassed both top-surface and cross-sectional morphology. Images of the film’s top surface were captured at 500× magnification, while images of the cross-section were captured at 1500×, 12,000×, and 30,000× magnifications.

A confocal microscope with white light lasers (STELLARIS 5, Leica, Germany) was used to examine the surface morphology and fluorescence properties of pure GRS, chitosan, and the GRS50/CS50 blend film. These samples, placed on glass slides, were observed at 63× magnification under a Transmitted Light Detector (TLD) and using a fluorescent contrasting method in their overlay channels. The resulting images present an overlay of fluorescence using excitation and emission wavelengths around 405 nm and 470 nm, respectively, consistent with the reported characteristics of the chitosan oligomer [24].

*Density*

The 1-inch diameter GRS/CS polymer films, punched into discs by a cork borer, were placed in a hot air oven at 105 °C for 24 h. The thickness of each film sample was measured using a Mitutoyo Thickness Gauge (Kawasaki, Kanagawa, Japan), while the weight was also recorded. The polymer film density (ρ) was calculated by dividing the weight (m) by the volume (v) using the following equation: ρ = m/v. Reported data represent the average of 3 samples per replicate across 3 replicates.

*Water solubility (WS)*

WS measurements were conducted on 1-inch diameter discs of GRS/CS polymer films, obtained by punching with a cork borer. The discs were subjected to 24 h treatment in a hot air oven at 105 °C, and the initial dry weight (W_initial_) of each disc was recorded. Subsequently, the dried films were immersed in 50 mL of distilled water in sealed beakers at room temperature for 24 h. After removing the film residues from the beakers, they were dried at 105 °C for 24 h and reweighed (W_final_) to determine the dry matter. WS of each film was calculated using Equation (2). The reported data represent the average of 3 samples per replicate across 3 replicates.

WS = ((W_initial_ − W_final_)/W_initial_) × 100 (2)

*Water contact angle*

The water contact angle, as an indicator of the film’s hydrophilic properties, was measured using a Data Physics Instruments OCA15 (GmbH, Filderstadt, Germany). Each film applied to the mango surface was tested in triplicate using the sessile drop observation. In this method, 1 μL droplets of distilled water were analyzed at various locations on the film’s surface.

*Mechanical properties*

The mechanical properties, including tensile strength and elongation, were assessed according to the standard test method of thin plastic sheeting [25]. Strips of GRS/CS films measuring 10 mm in width and 60 mm in length were prepared for testing. A Universal Testing Machine (Tinius Olsen, Horizon program, Salfords, Surrey, England) was utilized with an initial grip separation of 50 mm, an initial gauge length of 25 mm, and a probe speed of 50 mm/min. The reported data of each GRS/CS sample represent the average of 3 samples per replicate, with 3 replicates.

#### 2.2.2. Experiment Part II: Implementing GRS/CS Active Coatings with Varying Molar Ratios on Mango Surfaces

The maturity of mangoes was determined based on specific gravity [26]. Selected mature mangoes were washed with calcium hypochlorite solution (200 ppm), followed by rinsing with distilled water, and were then air-dried at room temperature for 45 min. Subsequently, the mangoes were immersed in different GRS/CS coating solutions (GRS100/CS0, GRS70/CS30, GRS50/CS50, GRS30/CS70, and GRS0/CS100) for 1 min and air-dried for 30 min at room temperature. The uncoated mangoes served as the control. The control and GRS/CS-coated mangoes were stored at room temperature for 10 days. The qualities of the mangoes, including appearance, total soluble solids (TSS), titratable acidity (TA), and the TSS/TA ratio, were evaluated at day 0 and at 2-day intervals over the 10-day storage period. Each treatment was replicated 3 times.

*Characterization of GRS/CS film coated on mango surface*

The adherence of the coating on the mango surface was assessed through stereo microscopic observation. Cross-sectional slices were obtained from the middle of the mango cheek using a stainless-steel blade for each treatment (GRS100/CS0, GRS70/CS30, GRS50/CS50, GRS30/CS70, and GRS0/CS100), as well as for the uncoated mangoes. The samples surfaces were examined at 45× magnification using a Leica Stereo Microscope (Model S Apo Stereozoom 1.0×–8.0×, Singapore).

The efficiency of polymer coating on mango surface and the interaction between chitosan and GRS were examined by analyzing the FTIR spectra of the samples. A Spectrum Two FTIR spectrometer ((PerkinElmer, Shelton, CT, USA)) equipped with an ATR sampling accessory was employed for this purpose. The infrared spectra of each treatment were recorded in 4000–400 cm^−1^, using 32 scans and a resolution of 4 cm^−1^.

#### 2.2.3. Statistical Analysis

The experiments were performed based on a completely randomized experimental design (CRD). Three replicates per treatment were applied in each experimental stage and data are expressed as mean ± standard deviation. The statistical comparisons of qualities of film, percent of mycelial inhibition, and qualities of coated mango were performed by one-way analysis of variance (ANOVA) using IBM SPSS Statistics 22 for Windows User (Chicago, IL, USA). The significance of differences among treatment means was compared by Duncan’s Multiple Range Test with 95% confidence level.

## 3. Result and Discussion

### 3.1. Part I: Effects of Varying Molar Compositions of GRS to Chitosan on GRS/CS Solution and Film Properties

#### 3.1.1. Physical and Chemical Properties of GRS/CS Blend Solutions and Films

From Table 1, it is evident that the pH decreases with the chitosan content. Chitosan, being insoluble in water, dissolves effectively in weak acid solvents such as formic, acetic, propionic, and lactic acids [27]. In this study, acetic acid was chosen as the solvent due to its safety for consumption and its ability to produce chitosan films with desirable properties. Compared to other weak acids, acetic acid yields films with low oxygen and water vapor permeability, hydrophobicity, mechanical strength, and excellent clarity [27], making it ideal for coating or packaging fruits and vegetables intended for consumption.

However, a notable limitation of using chitosan films prepared with acetic acid as a coating or packaging material for food products is the persistent pungent odor and sour taste of acetic acid, which remains in the film and cannot be entirely eliminated. This odor and taste can transfer to the food or produce coated with the film, potentially affecting consumer acceptance [28].

To mitigate these limitations, diluting chitosan with GRS can be employed to reduce the concentration of acetic acid in the film. This approach results in a film with modified properties that are better suited for use as a coating or packaging material to extend the shelf life of food, vegetables, and fruits while minimizing undesirable sensory effects.

The viscosity of the coating solution plays a critical role in determining film formation and performance, thereby influencing its efficacy for active coating applications on produce. It directly affects film thickness and uniformity on surfaces, while also playing a significant role in determining the film’s barrier properties against gases and water vapor. Therefore, understanding the viscosity of the coating solution is important for optimizing the functional properties of resulting films and ensuring their efficacy in packaging.

From Table 2, the viscosity analysis revealed that the pure GRS solution exhibited a viscosity of 27.3 cp, markedly lower than the pure chitosan solution at 667.5 cp, consistent with prior research [29]. This pronounced disparity in viscosities indicates that using GRS alone may not be optimal for its intended application as a coating to extend the post-harvest shelf life of fruits and vegetables. The low viscosity of the GRS solution could hinder its ability to form a cohesive film on produce surfaces, potentially compromising its effectiveness in preserving quality. This lower viscosity of GRS can be attributed to its composition, characterized by a low amylose content (1–2%) and a high amylopectin content (98–99%) [30]. Amylopectin, being highly branched with (1→4)-linked α-*D*-glucosyl units in chains connected by (1→6) linkages (4–5%), exhibits a semi-crystalline nature [31]. In the case of GRS, amylopectin consists of shorter chains (19–20 glucose units), limiting its capacity to form entanglements and consequently reducing viscosity. Conversely, chitosan, with its linear chains composed of long segments exceeding 6200 units of *N*-acetylglucosamine and glucosamine, can form numerous entanglements, resulting in higher viscosity. Blending GRS or another waxy starch with chitosan increases viscosity of the starch due to several factors. Chitosan can form hydrogen bonds with starch molecules, leading to a network structure and increased viscosity [32].

Additionally, the introduction of chitosan into the GRS solution creates more entanglements between polymer chains, further increasing viscosity. The enhanced viscosity promotes better film formation on produce surfaces, leading to improved adhesion and coverage, thereby enhancing the effectiveness of the coating in extending the shelf life of produce. An exponential increase in viscosity observed in GRS/CS blend solutions with higher chitosan concentrations suggests a significant role of intermolecular hydrogen bonding between starch and chitosan. This phenomenon likely contributes to the enhanced interactions within the polymer matrix, leading to greater viscosity. Consequently, the viscosity of the polymer blend solution can be adjusted as needed to suit the intended use by modifying the proportions of GRS and chitosan. Notably, the viscosity of the polymer solutions significantly influences the adhesion and thickness of the polymer coating on fruit and vegetable surfaces. This observation aligns closely with the experimental results obtained from employing GRS/CS blend solution as a coating to extend the shelf life of Nam Dok Mai mangoes in this research, in that the film thickness increased notably with higher chitosan proportion.

The optical properties of a polymer play a vital role in determining its suitability and performance for preserving the quality and marketability of produce. A coating with desirable optical characteristics enhances the visual appearance, facilitates quality control, and boosts consumer confidence, ultimately contributing to the success of the product in the marketplace. Table 2 provides valuable insights into the optical properties of the films, particularly the mixed GRS/CS solution with high chitosan content, which exhibits a viscosity suitable for coating fruits and vegetables. However, there are color limitations to consider, as pure chitosan film tends to have a slight yellowish tint attributed to carotenoid pigments, primarily astaxanthin, which forms strong bonds with the chitin molecule and interact with proteins in the exoskeleton’s epithelial layer [33]. Rigorous chemical treatment is required to produce colorless chitosan products, albeit compromising some properties [33]. This yellow hue may pose limitations when used as a coating or wrapping, distorting product color and potentially influencing consumer decisions. Conversely, pure GRS film exhibits clarity without coloration. Blending with colorless GRS reduces the yellow tint of the chitosan film, with the GRS/CS film showing a moderate yellow hue due to dilution effect, while optical clarity remains consistent across varying ratios. This underscores the remarkable amorphicity of both pure and blended polymer films, making GRS/CS blend film more suitable for coating or wrapping. Despite the yellow tint in films with high chitosan content, they maintain aesthetic appeal enhancing overall appearance as observed in the mango coating study.

The density of the polymer coating affects its ability to act as a barrier against external factors, such as moisture, gases, and contaminants. This property is crucial for extending the shelf life of produce by minimizing moisture loss and preventing the ingress of oxygen, which can accelerate deterioration. The density of the chitosan film, measured at 1.09 g/cm^3^, aligns with the existing literature [34] and falls below that of the GRS film at 1.88 g/cm^3^, similar to high amylopectin corn starch film at 1.74 g/cm^3^ [35]. As the starch content increases in GRS/CS blended films, the density shows a linear rise attributed to amylopectin molecules integrating into the interstitial spaces of chitosan chains. This incorporation of glutinous rice starch in the GRS/CS blend fosters a more compact arrangement of polymer chains within the film matrix, resulting in enhanced packing density and, consequently, increased film density. Moreover, interactions, particularly hydrogen bonding between chitosan and GRS molecules, facilitate the formation of a dense and cohesive film structure.

An increase in chitosan content leads to decreased water solubility, reflecting its intrinsic hydrophobic nature. This is evidenced by the water contact angle of the films, which is further discussed. Consequently, blending chitosan with GRS enhances various physical properties of the film, including optical characteristics, density, and hydrophobicity, rendering it more suitable for specific applications. By adjusting the proportions of GRS and chitosan, the properties of GRS/CS blend films can be finely adjusted to meet distinct application requirements. This blending strategy not only improves the film’s physical attributes but also enables the optimization of properties tailored to specific applications. Thus, the integration of chitosan with GRS offers a versatile approach for enhancing film properties and broadening its potential utility across diverse applications.

#### 3.1.2. Effect of GRS/CS Coating Solution on *C. gloeosporioides* Mycelial Growth

The *C. gloeosporioides* is a prevalent plant pathogen found worldwide, notably thriving in tropical and subtropical climates. It causes anthracnose disease across a diverse array of crops, including almond, avocado, apple, coffee, guava, mango, strawberry, papaya, banana, passion fruit, citrus, grapes, and cashews [36]. Anthracnose represents a significant threat to mango production worldwide, affecting both the economic viability of mango farming and the availability of high-quality mangoes for consumers [37].

**Figure 1:**
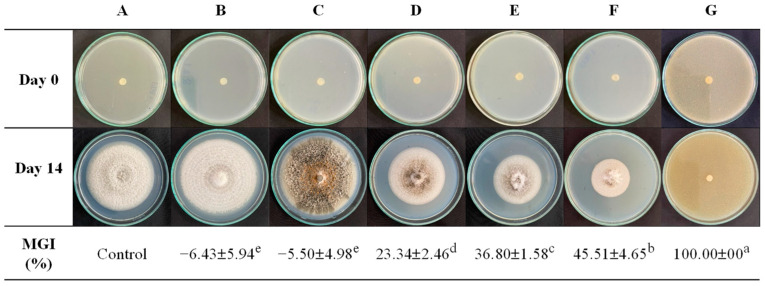
Effect of coating solution on *Colletotrichum gloeosporioides* mycelial growth and the percent of mycelial growth inhibition; control (**A**) (without coating solution in PDA), 0.1% acetic acid added (**B**), coating solution added in PDA: GRS100/CS0 (**C**), GRS70/CS30 (**D**), GRS50/CS50 (**E**), GRS30/CS70 (**F**), and GRS0/CS100 (**G**). Footnote: Values are presented as mean ± standard deviation. Values with the same superscript are not significantly different (*p* ≥ 0.05).

This study investigated the impact of the molar ratio of GRS/CS on its inhibitory efficacy against *C. gloeosporioides* strains isolated from mangoes. The mycelial growth inhibition (MGI) of coating solution by radial growth on PDA after incubation for 14 days is shown in Figure 1. The results reveal that the pure GRS solution facilitated extensive fungal growth, surpassing even the control group, as starch serves as a nutrient source for fungi and other microorganisms. Consequently, pure starch (GRS100/CS0) is unsuitable for food or fruit coatings or packaging due to its susceptibility to microbial proliferation. However, antibacterial properties were contributed by the chitosan admixture. MGI values for GRS/CS blends notably increased with rising chitosan content (*p* < 0.05), with complete inhibition observed for the pure chitosan (GRS0/CS100). This underscores chitosan’s efficacy in anthracnose inhibition, a pivotal factor in mango deterioration globally. Previous studies have supported chitosan’s efficacy, with concentrations of 1.5–2% yielding over 70% inhibition of *C. gloeosporioides* [38,39]. Furthermore, chitosan effectively inhibited *C. gloeosporioides* development in other crops, such as papaya [40] and manila mango [41]. Chitosan’s antifungal effects depend on electrostatic interactions between its protonated amino groups and the negatively charged phospholipids of fungal membranes [42]. Chitosan induces membrane permeabilization, disruption, and release of cellular contents. Additionally, chitosan can penetrate fungal cells, inhibiting DNA/RNA synthesis, disrupting protein synthesis, and altering gene expression [43].

#### 3.1.3. Morphology of the GRS/CS Blend Films

The structure and morphology of a polymer blend film strongly influence its mechanical and barrier properties, which in turn are crucial aspects for its use as a coating or packaging material to extend the shelf life of agricultural and food products. Therefore, analyzing these aspects provides valuable insights into the film’s performance and suitability for various applications.

**Figure 2:**
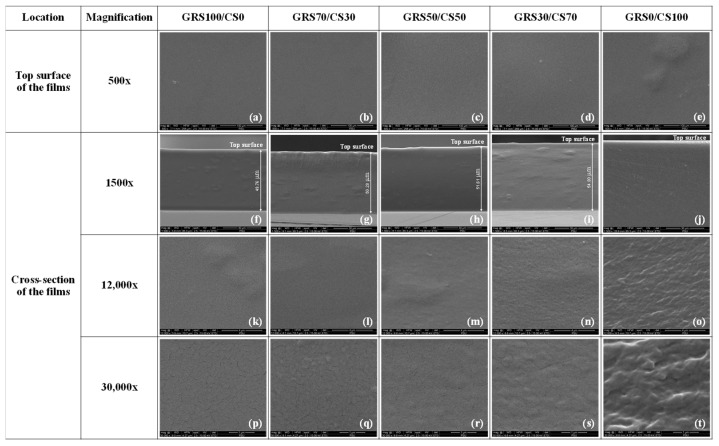
SEM micrographs of GRS/CS blend films with varying molar proportions. Top surfaces of the films at 500× magnification, scale bar: 100 µm (**a**–**e**). Cross-sections of the films at 1500× magnification, scale bar: 30 µm (**f**–**j**). Cross-sections of the films at 12,000× magnification, scale bar: 4 µm (**k**–**o**). Cross-sections of the films at 30,000× magnification, scale bar: 1 µm (**p**–**t**).

SEM images were utilized to examine the surface characteristics of pure GRS, chitosan, and GRS/CS films at different blend ratios (Figure 2a–e). The images reveal smooth surfaces across all compositions, indicating excellent compatibility and no microphase separation. Cross-sectional analysis at 1500× (Figure 2f–j) unveiled thickness variations corresponding to film density reduction with increasing chitosan content, consistent with Table 2. At higher magnifications of 12,000× (Figure 2k–o) and 30,000× (Figure 2p–t), the pure starch film (GRS100/CS0) displayed numerous cracks, indicative of its inherent brittleness. These cracks can be attributed to various factors, including the formation of dense and rigid structures within the film matrix due to the waxy starch’s high amylopectin content. Moreover, the drying process and non-uniform distribution of starch particles contribute to crack formation [44]. These cracks significantly impact the mechanical and barrier properties of the GRS film, rendering it inadequate for coating or packaging perishable produce. However, the incorporation of chitosan mitigates crack formation, with an increase in chitosan content corresponding to a reduction in crack prominence and improvement in mechanical properties. Furthermore, the presence of chitosan in the film coating applied to mango fruit may reduce the diffusion of water vapor and oxygen gas, thereby delaying mango ripening and minimizing the appearance of shriveling.

Pure chitosan films display a characteristic corrugated appearance under SEM analysis, characterized by uniform density devoid of cracks or air bubbles (Figure 2o,t), in contrast to GRS-mixed films. This uniform morphology correlates with the improved mechanical and barrier properties observed for chitosan films. The corrugated appearance may be attributed to chitosan’s linear heteropolysaccharide structure and its interactions with neighboring molecules, resulting in irregular arrangements within the film matrix. Furthermore, structural modifications occurring during film formation contribute to this distinctive morphology [45].

Fluorescence microscopy is a valuable tool for assessing the miscibility and morphology of polymer blends. By labeling specific components with fluorescent dyes or probes, it enables visualization of polymer distribution, enhancing our understanding of blend structure and performance. However, a limitation is the need for suitable fluorescent labels or probes, which may affect observed morphology and lead to misinterpretation [46]. In our study, we present a novel approach utilizing fluorescence microscopy to examine the miscibility and morphology of the GRS/CS composite film, offering an initial evaluation of its applicability for amine-containing polymers without the use of dyes or fluorescent probes.

**Figure 3:**
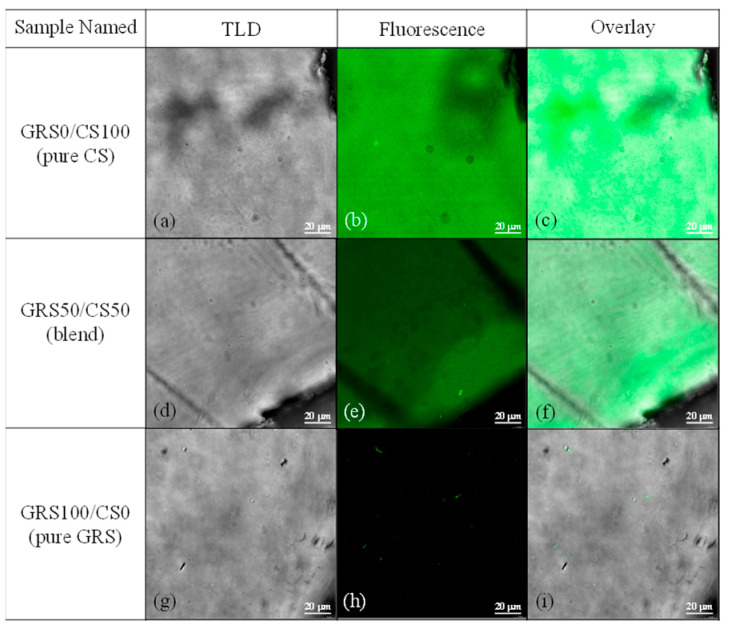
Microscope images of pure chitosan (**a**–**c**), pure GRS (**g**–**i**), and the GRS50/CS50 blend film (**d**,**e**,**f**). The sample films’ top surfaces captured at 63x magnification, scale bar: 20 µm, using different modes: Transmitted Light Detector (TLD) (**a**,**d**,**g**), fluorescence (**b**,**e**,**h**), and an overlay of TLD and fluorescence (**c**,**f**,**i**).

Despite lacking typical fluorescence-associated structures, chitosan exhibited fluorescence, particularly in its oligomeric form [24]. Studies suggest that certain polymers with amine groups can display fluorescence due to the reaction between amine groups and CO_2_, forming fluorescent carbamato anions (NHCOO^−^) [24]. The amino groups of chitosan interact with airborne CO_2_, generating fluorescent carbamato anions, with observed excitation and emission wavelengths at approximately 400 nm and 470 nm, respectively [24]. Fluorescence intensity is directly correlated with the concentration of chitosan oligomers [24]. Microscopic analysis of the pure chitosan film (Figure 3a,c) revealed a distinctive corrugated pattern accompanied by consistent fluorescence throughout (Figure 3b). In contrast, the pure GRS film (Figure 3g,i) displayed a uniform appearance without fluorescence (Figure 3h) due to the absence of fluorescence chromophores and amine groups in its structure. The minimal fluorescence observed in Figure 3h may be attributed to proteins on the microbial cell surface, where amino acids react with atmospheric CO_2_, akin to the behavior observed in chitosan.

Microscopic images of the GRS50/CS50 blend film (Figure 3d) showed a uniform appearance similar to the pure GRS film. The fluorescence image (Figure 3e) and overlay image (Figure 3f) of the blend film confirmed excellent compatibility with no phase separation at the microscopic level. The decreased fluorescence intensity observed in the blend film (Figure 3e), relative to the pure chitosan, indicates a lower concentration of glucosamine units in the polymer composite. This is likely due to a dilution effect. This finding reinforces the idea that changes in chitosan content directly influence fluorescence properties, emphasizing the successful blending and compatibility of GRS and chitosan within the composite film structure. 

#### 3.1.4. Water Contact Angle of GRS/CS Film

Hydrophobicity plays a crucial role in determining the essential properties of active coatings aimed at extending the shelf life of produce, thereby preserving their freshness and quality. It exerts a profound influence on key characteristics including water vapor barrier properties [47,48], microbial growth inhibition [47], adhesion to produce surfaces [48], and gas permeability [47,48]. Striking the right balance among these properties is crucial for developing effective coatings that can maximize the shelf life and quality of fruits and vegetables [48]. Hence, this study investigates the water contact angle to elucidate the hydrophobic nature of the films. Additionally, the effect of the molar ratio between GRS and chitosan on the hydrophobicity of GRS/CS composite films is explored, aiming to identify compositions suitable for various applications.

**Figure 4:**
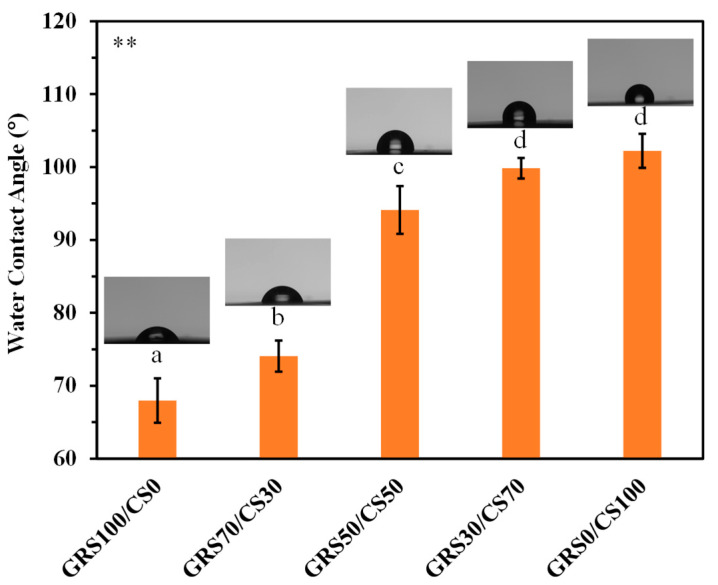
The water droplet profiles and the water contact angles for pure GRS, pure chitosan, and the GRS/CS blend films with different molar proportions coated on the mango surface. Footnote: ** *p* ≤ 0.01 differences are highly significant, different letters above the bars indicate statistically significant difference.

The water contact angles provide insight into the influence of the polymer composition on the hydrophobicity of GRS/CS composite films, shown in Figure 4. The pure GRS film displays high hydrophilicity, characterized by a low contact angle of 68°, primarily attributed to the abundance of hydroxyl groups in starch. This hydrophilic nature of starch coatings predisposes them to water absorption, potentially enhancing moisture permeation, which could accelerate spoilage [49]. Furthermore, when using starch films under high-humidity atmospheres, such as coatings for fruits and vegetables, the heightened hydrophilicity may elevate gas permeability, thereby impacting gas-exchange dynamics and influencing the produce deterioration rate [50]. Consequently, the hydrophilic GRS film is considered unsuitable for applications as coatings to extend the shelf life of post-harvest produce.

In contrast, the pure chitosan film exhibited the highest hydrophobicity, with a contact angle of 102°, attributed to the presence of hydrophobic acetyl groups in incompletely deacetylated chitosan [51]. This hydrophobic characteristic contributes to excellent water vapor barrier properties, effectively preventing produce dehydration and maintaining optimal texture and appearance over extended periods [51]. Additionally, in high-humidity atmospheres, the hydrophobic coatings typically demonstrate low gas permeability, facilitating controlled gas exchange of oxygen and carbon dioxide [50]. This regulation of gas exchange helps manage respiration rates in produce, thereby slowing down the ripening process and extending shelf life. Moreover, hydrophobic coating acts as a barrier against moisture, inhibiting the growth of microorganisms like bacteria and fungi [47]. These findings are corroborated by a 10-day storage study, which underscores the efficacy of chitosan coatings in delaying ripening by mitigating fruit transpiration.

The hydrophobicity of the GRS film can be enhanced by blending with chitosan, and the hydrophobic nature of GRS/CS composite films is improved with increased chitosan content. Consequently, the hydrophobicity of the GRS/CS coating can be readily tailored to accommodate the respiration rate and dehydration characteristics of each type of produce, thereby preserving produce quality for an extended duration. These findings are consistent with those of a 10-day storage study, wherein higher chitosan content resulted in prolonged ripening delay and reduced shriveling appearance of mango fruits.

#### 3.1.5. Mechanical Properties of the GRS/CS Film

The mechanical properties of the active coating polymer significantly influence its ability to provide durable, flexible, and effective protection to produce against physical damage, microbial contamination, and environmental factors in general [52]. Optimizing these properties is crucial for developing coatings that meet the specific requirements of different types of produce and storage conditions, ultimately enhancing their shelf life and marketability.

**Figure 5:**
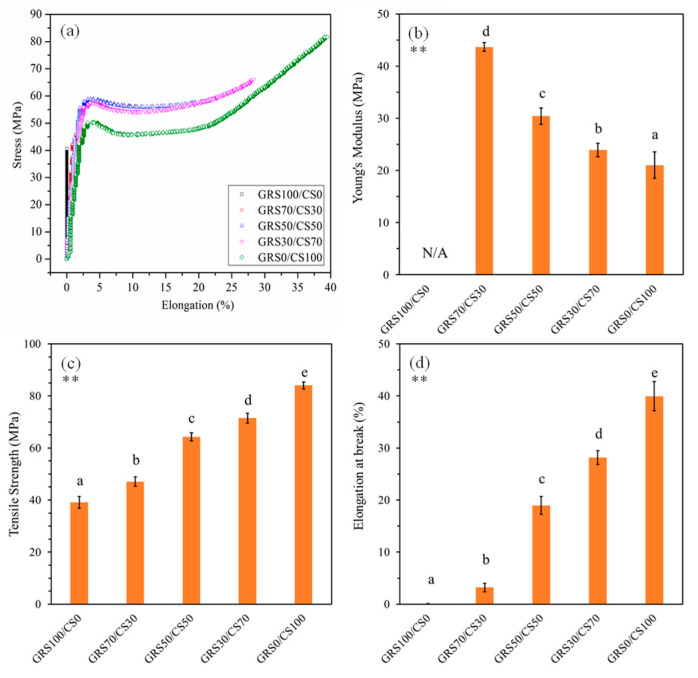
Stress–strain curves (**a**), Young’s modulus (**b**), tensile strength (**c**), and elongation at break (**d**) of the GRS/CS blend films with different molar proportions. Footnote: ** *p* ≤ 0.01 differences are highly significant, different letters above the bars indicate statistically significant difference.

Analysis of the stress–strain curves depicted in Figure 5a reveals distinct mechanical properties of the films. The GRS film exhibits characteristics of rigid and brittle fracture, attributed to the robust hydrogen bonding present in the polysaccharide chains, as well as its specific composition and molecular structure [53]. GRS, predominantly composed of amylopectin with minimal amylose content, demonstrates a semi-crystalline nature [54]. This molecular arrangement fosters tightly packed structures within the film matrix, resulting in reduced flexibility and limited mobility of the polymer chains, thereby displaying rigidity and brittleness during mechanical testing [53]. Conversely, the chitosan film shows a ductile response with a notable plastic range [55], making it suitable for applications requiring flexibility and strength, such as produce coatings.

Blending GRS with chitosan enhances the mechanical properties of starch films, rendering them suitable for use as active coatings for produce. The mechanical properties of GRS/CS blend films can be tailored for specific applications by adjusting the proportions of starch and chitosan. Increasing chitosan content improves flexibility and strength, resulting in films with enhanced adhesion to produce surfaces, resilience to temperature changes, resistance to physical damage, and maintenance of barrier integrity against moisture, gases, and microbes. Films with a higher starch content, such as the 70GRS/30CS blend, retain some brittleness but exhibit improved tensile strength and elongation compared to pure GRS. Conversely, films with more chitosan, such as the 50GRS/50CS and 30GRS/70CS blends, demonstrate ductile behavior similar to pure chitosan. Tensile strength and elongation increase, while modulus decreases with chitosan content, highlighting the tunability of mechanical properties in GRS/CS blend films.

### 3.2. Part II: Implementing GRS/CS Active Coatings with Alternative Molar Ratios on Mango Surfaces

#### 3.2.1. Characterization of GRS/CS Coating on Mango Surface

**Figure 6:**
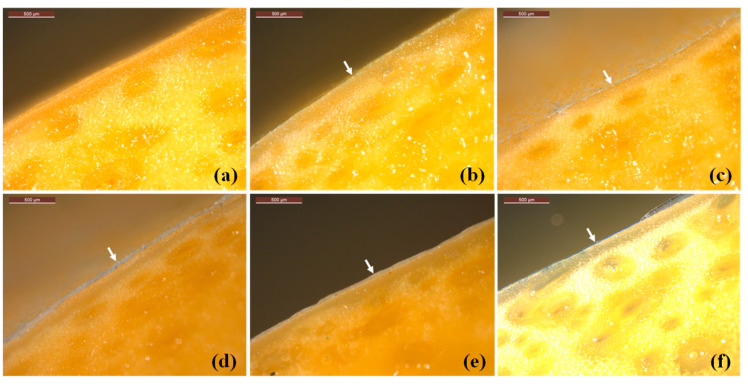
Optical stereo microscope images of cross-sections: film free mango surface (**a**), and the mango surfaces coated with GRS100/CS0 (**b**), GRS70/CS30 (**c**), GRS50/CS50 (**d**), GRS30/CS70 (**e**), and GRS0/CS100 (**f**). The scale bar is 500 µm. The white arrow indicates the GRS/CS film coated on the mango surface.

This experimental section aims to evaluate the efficacy of GSR/CS as an active coating and investigate the impact of the starch to chitosan ratio on the suitability of polymer blend as a coating for maintaining post-harvest mango quality. This section places particular emphasis on critical parameters such as film thickness and adhesion to the mango surface. These factors play crucial roles in determining the efficacy of an active coating in preservation of post-harvest produce, contributing to the establishment of a robust barrier against external factors, ensuring uniform protection and enhanced durability during handling and storage. In Figure 6, the mango cross-section exhibits a uniformly coated surface throughout the polymer film, clearly indicated by the white arrow. Conversely, Figure 6b depicts a thinly coated mango surface with the GRS film, barely visible due to several contributing factors. The low viscosity of the GRS coating solution, coupled with its high film density and hydrophilic nature, leads to weak interaction with the hydrophobic cutin compounds present on the mango surface, resulting in poor adhesion. Thus, GRS alone proves inadequate as a coating for extending the fruit’s shelf life.

Blending GRS with chitosan significantly enhances film adhesion and thickness on the mango surface, as observed in our experimental findings. The thickness of the GRS/CS film coating increases significantly with chitosan content. This trend is particularly evident in the pure chitosan film, which emerges as the thickest among all coatings. Figure 6f illustrates the film with a thickness of approximately 10 µm on the mango surface, clearly visible under the microscope and shown in the image with a white arrow. This can be attributed to chitosan’s hydrophobic nature, fostering strong interaction with the hydrophobic cutin compounds found in mango lenticels—macroscopic openings, approximately 0.2 mm in size, formed during fruit growth when stomata rupture [56]. Mango lenticels primarily comprise natural wax, facilitating gaseous exchange and transpiration. The robust adhesion of chitosan to mango lenticels ensures the coating’s integrity, effectively protecting against physical damage and microbial contamination [57]. Conversely, hydrophilic coatings like starch may exhibit weaker adhesion to fresh produce surfaces, potentially compromising freshness preservation. Therefore, GRS/CS polymer composite films with higher chitosan content are more likely to be produce effective coatings. Adjusting the starch to chitosan ratio allows us to fine-tune film thickness and coating efficiency to suit different produce types.

**Figure 7:**
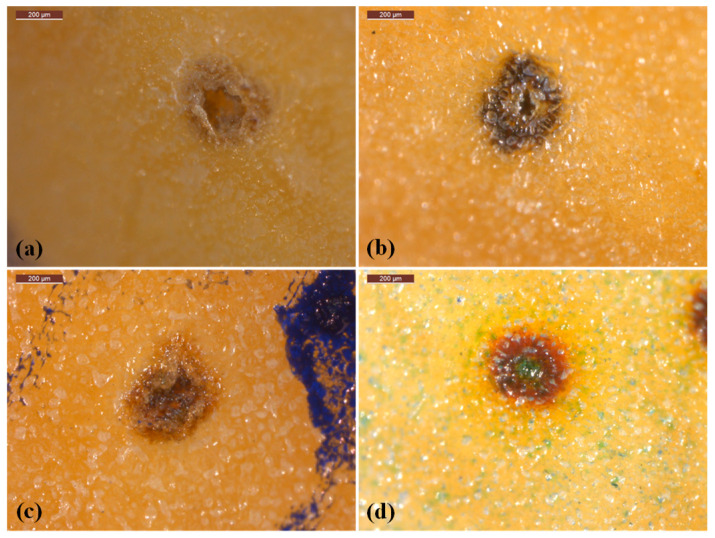
Optical stereo microscope images of lenticels on film free mango surface (**a**), and on mango surfaces coated with GRS100/CS0 (**b**), GRS50/CS50 (**c**), and GRS0/CS100 (**d**). The scale bar is 200 µm.

The experimental findings align with previous studies on the efficacy of polymer film coatings on mango lenticels. Figure 7a illustrates the lenticels on the free surface of mangoes, revealing varied performances of polymer coatings in covering these structures. This divergence can be attributed to the hydrophobicity and viscosity of the polymer solutions. The GRS coating, characterized by low viscosity and high hydrophilicity, only partially covers the lenticels, as depicted in Figure 7b. Consequently, the pure GRS film proves inadequate in effectively controlling gas and water vapor diffusion, failing to delay produce ripening and to reduce shriveling. In contrast, the hydrophobic nature of chitosan enables effective coverage of mango lenticels, as depicted in Figure 7d.

Blending GRS with the hydrophilic chitosan, which also exhibits high viscosity, enhances lenticel coverage, as demonstrated in Figure 7c. This renders the GRS/CS blend film more suitable for use as a produce coating. These findings are consistent with FTIR analysis and a 10-day storage study, indicating that chitosan coating delays mango ripening by reducing respiration and alleviates wilting by minimizing transpiration. In contrast, the GRS coating, which incompletely covers the lenticels, lacks efficiency in delaying ripening and reducing shriveling.

**Figure 8:**
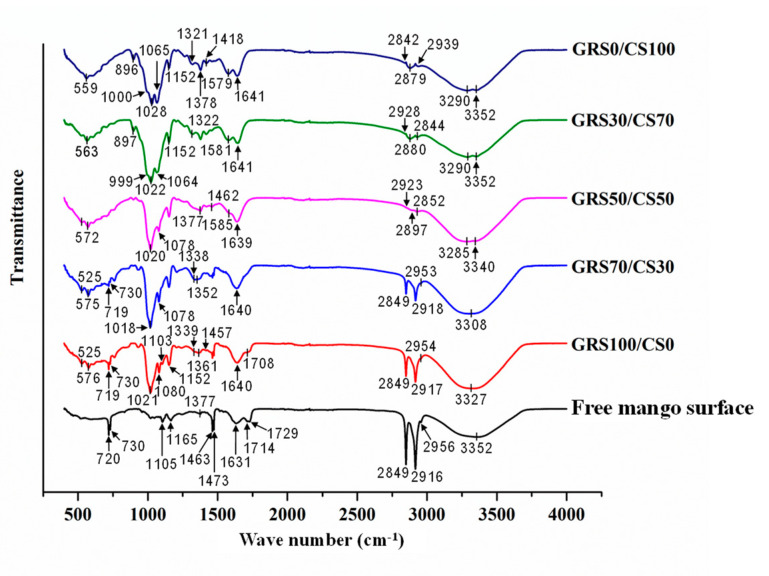
FTIR spectra of the film free mango surface compared with mango surfaces coated with pure GRS (GRS100/CS0), pure chitosan (GRS0/CS100), and the GRS/CS blend films with different molar proportions.

This study effectively utilized ATR-FTIR spectroscopy to assess the efficiency and effectiveness of polymer film coatings on produce or food surfaces, offering a valuable analytical tool within food science and technology. By employing FTIR spectroscopy, we gained insights into the chemical composition, molecular structure, and interaction of the polymer film with the surface of the produce or food. Our findings underscore the potential of FTIR spectroscopy as an effective method applicable to various food and product-related analyses. Figure 8 shows the FTIR spectra of the pure polymers and GRS/CS blended polymer films with different molar compositions coated on mango surface compared to the spectra of uncoated mango surface. The characterization of uncoated mango surface by FTIR spectroscopy has provided significant information on the nature of functional groups present in the cuticle matrix.

A broad band around 3352 cm^−1^ assigned to the stretching of hydroxyl (O-H) groups mainly contributed by the polysaccharide and the non-esterified hydroxyl groups of cutin [58]. The asymmetric stretching of CH_3_ groups was found at 2956 cm^−1^, and the asymmetric and symmetric stretching of CH_2_ groups were found at 2916 and 2849 cm^−1^, respectively. The CH_2_ scissoring at 1473 and 1463 cm^−1^, as well as the CH_2_ rocking at 730 and 720 cm^−1^, were attributed to aliphatic compounds, i.e., cutin, waxes, and cutan, in the plant cuticle [59]. The C=O stretching at 1729 cm^−1^ and the shoulder at 1714 cm^−1^, as well as the asymmetric and symmetric C-O-C stretching at 1165 and 1105 cm^−1^, were associated with ester and carboxylic acid groups of the cutin matrix [60].

The FTIR spectrum of mango surface coated with pure GRS film (GRS100/CS0) still exhibits characteristic peaks of the mango cutin layer at 2916, 2849, 1473, 1463, 730, and 720 cm^−1^. This indicates that the GRS film covers the mango surface partially or in certain areas. Additionally, the spectrum displayed characteristic peaks of the GRS, including a broad band at 3327 cm^−1^ attributed to the stretching vibration of hydrogen-bonded hydroxyl groups (O-H) [61]. Another distinctive peak at 1640 cm^−1^ was assigned to the O-H stretching of hydroxyl groups [62]. The spectrum also exhibited noticeable absorbances at 1152, 1103, 1080, and 1021 cm^−1^, which can be attributed to C-O and C-C stretching with some contribution from C-OH [63].

Coating mango surfaces with a GRS/CS polymer film with an increased proportion of chitosan showed an improved tendency to adhere and cover the mango surface effectively. This was evident from the intensity of the characteristic peaks of the cutin layer at 2916, 2849, 1473, 1463, 730, and 720 cm^−1^, which decreased as the quantity of chitosan in the coating increased. The GRS/CS polymer film achieved complete coverage of the mango surface when the chitosan content in the coating reached 50% by moles. This is observed in the absence of the cutin peaks in the FTIR spectrum of mango coated with GRS50/CS50, GRS30/CS70, and pure chitosan (GRS0/CS100) films. In addition to the decrease in intensity of the cutin peaks as described above, a minor shift in the peak positions was also observed. The asymmetric stretching of CH_3_ groups at 2956 cm^−1^ and CH_2_ rocking at 720 cm^−1^ showed a slight decrease in wavenumber, while the asymmetric stretching of CH_2_ groups at 2916 cm^−1^ exhibited a slight increase in wavenumber, corresponding to the increase in chitosan content in the coating. These FTIR results agree well with the microscope images shown in Figure 6.

The polymer coating adhesion to the mango surface is attributed to the interaction between the hydroxyl and carboxylic acid groups of the cutin layer and the active hydroxyl groups of GRS and chitosan. This is supported by the observed peak shifts of the hydroxyl and carboxylic acid groups of the cutin layer when coated with pure GRS. For instance, the stretching of hydroxyl groups of cutin shifted from 3352 to 3327 cm^−1^, and the C=O stretching of carboxylic acid groups of cutin shifted from 1729 and 1714 cm^−1^ to 1708 cm^−1^, indicating these interactions. These peak shifts towards lower wavenumbers reflect the stronger bonding between the hydroxyl and carboxylic acid groups of the cutin layer, which is further enhanced by increasing the amount of chitosan in the coating. Coating with pure chitosan was found to completely cover the mango surface, as confirmed by the absence of cutin peaks in the FTIR spectrum of GRS0/CS100 sample. Instead, characteristic peaks associated with chitosan were observed. These include the broad bands at 3352 and 3290 cm^−1^ corresponding to N-H and O-H stretching, respectively [64]. The absorption bands at 2939 and 2879 cm^−1^ correspond to C-H symmetric and asymmetric stretching, respectively, which are associated with the vibration of the characteristic pyranose ring of polysaccharides [65]. Residual *N*-acetyl groups were identified by the band at 1641 cm^−1^ (C=O stretching of amide I), while the band at 1579 cm^−1^ corresponds to N-H bending of the primary amine [66]. CH_2_ bending and CH_3_ symmetrical deformations were confirmed by bands at 1418 and 1378 cm^−1^, respectively [67]. The band at 1152 cm^−1^ is assigned to the asymmetric stretching of the C-O-C in glycosidic linkage, while C-O stretching for primary alcohol was observed at 1065 and 1028 cm^−1^ [62]. The band at 896 cm^−1^ belongs to CH out-of-plane bending vibration in the monosaccharide ring [67].

Comparing the FTIR spectra of mango surfaces coated with pure chitosan film (GS0/CS100) and the GRS/CS polymer blend films, it was observed that the presence of GRS in the coating led to increased freedom and disorder of the chitosan chains. This can be seen from the shift in peaks associated with the vibration of the pyranose ring towards higher wavenumbers as the GRS content increased. It is speculated that the interaction between chitosan, GRS, and mango cutin occurs primarily through the hydroxyl groups of chitosan. This is evident from the shifts in the O-H stretching at 3352 cm^−1^ and the C-O stretching at 1065 and 1028 cm^−1^ towards lower wavenumbers with increasing GRS content.

#### 3.2.2. Quality Aspects of the Mango Coated with GRS/CS

**Figure 9:**
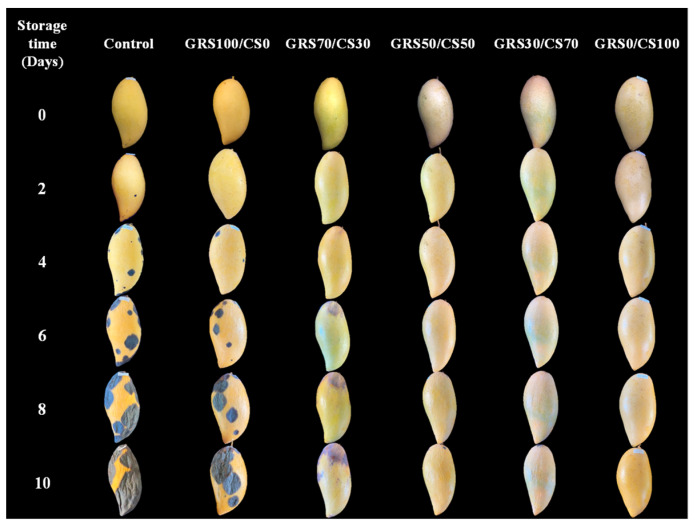
The appearances of mangoes without and with the experimental GRS/CS coatings during storage at room temperature for 10 days.

Mango samples, treated with film coatings having various GRS/CS ratios, were stored at room temperature for 10 days, with monitoring of physical appearance and TSS/TA every 2 days (Figure 9). Initially, all the samples exhibited firm skin without disease symptoms. The control and GRS100/CS0 samples developed significant anthracnose symptoms, expressed as dark, sunken lesions on days 2 and 4, respectively, consistent with observations by Hadthamard et al. [68] of uncoated samples. The GRS70/CS30 showed symptoms of stem end rot on day 6. These post-harvest diseases typically manifest several days after fruit collection, significantly influencing consumer decisions. This study affirms that coating with chitosan mole fraction from 50% up effectively prevents disease throughout storage. These findings align with previous observations, indicating enhanced inhibition of *Colletotrichum* spp. with increasing chitosan proportions. All GRS-CS treatments satisfactorily reduced dehydration and delayed mango ripening. Coated samples demonstrated less shriveling and color change than the control samples, attributed to the hydrophobicity of chitosan (high contact angle), which effectively covered lenticels and limited water migration. The primary function of the coating material is to reduce moisture and gas permeation between fruit and its environment, resulting in lower respiration rates, reduced water migration, and delayed ripening [69].

**Figure 10:**
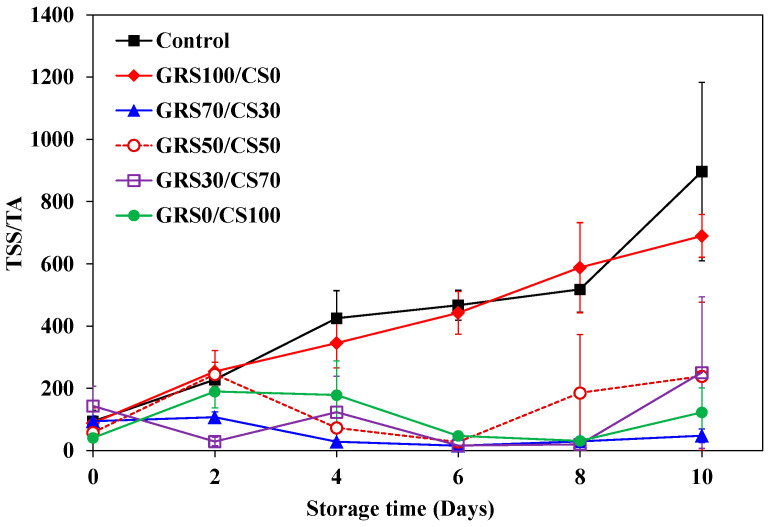
TSS/TA ratio for mangoes without and with the experimental GRS/CS coatings during storage at room temperature for 10 days. Vertical bars represent standard deviations.

During ripening, mangos undergo both physical changes, such as changes in peel and flesh color and loss of tissue firmness, as well as chemical changes, including heightened aroma, increased nutritional value, reduced acidity, and higher sugar content. Gas concentrations, particularly of oxygen (O_2_) and carbon dioxide (CO_2_), play a pivotal role in fruit ripening by influencing respiration rates. Effective gas control through the barrier properties of coating materials stands as a crucial solution for extending shelf life. Polysaccharide coating forms a tightly packed hydrogen-bonded network and serves as an effective oxygen barrier [70]. The application of chitosan coating successfully reduces the respiration rate and retards the ripening of mango and avocados [38,71]. Total soluble solids (TSS) relate to fruit sugar content, increasing as starch converts to sugar during ripening. On the other hand, titratable acidity (TA) indicates fruit acidity, decreasing with ripening. The ratio of TSS and TA is widely used to represent fruit taste, as a greater ratio means the fruit is sweeter and less sour. Initially, all treatments had TSS of 14–16°Brix and TA of 0.2–0.4%. The TSS/TA ratio (Figure 10) showed two distinct patterns: non-chitosan (control and GRS coating) and chitosan coating (various chitosan fractions). The non-chitosan group displayed a typical increasing TSS/TA pattern during storage, indicating ongoing ripening, with control and GRS100/CS0 reaching 896.5 and 689.6, respectively, after 10 days. Conversely, the chitosan-coated group exhibited a stable TSS/TA trend, signifying constant ripening. Chitosan-coated mangoes had TSS/TA in the range of 50–250, indicative of a turning ripened stage for Nam Dok Mai mango [72]. This finding demonstrates the efficacy of chitosan coating in delaying mango ripening. Similarly, other mango varieties (Tommy Atkins and Apple) exhibited delayed ripening when treated with 2% chitosan. Silva et al. [73] proposed that chitosan coating delays sugar accumulation and starch conversion. The findings of this study validate the efficacy of GRS-CS coating in extending the shelf life of mangoes. Uncoated mangoes met selling standards for 2 days, while those treated with at least 50% chitosan in coating maintained their quality throughout the entire 10-day storage period.

## 4. Conclusions

This study highlights the potential of GRS/CS blends as natural active coatings for extending mango shelf life, with properties adjustable by varying the molar ratio of GRS to chitosan. This blending strategy presents a versatile approach for enhancing film properties across diverse food industry applications. The results show that pH decreased with chitosan content in GRS/CS blend solutions due to acetic acid solvent for chitosan dissolution. To address odor and taste limitations of acetic acid, blending chitosan with GRS was proposed to dilute acetic acid concentration in the film. Moreover, blending chitosan with pure GRS increased viscosity, facilitating film formation on produce surfaces and improving coating effectiveness. GRS/CS blend films with higher chitosan content exhibited higher density, increased hydrophobicity, and improved mechanical properties, contributing to better moisture, gas, and contaminant barrier properties and inhibiting microbial growth. Chitosan’s hydrophobic nature fostered strong interaction with mango lenticels, ensuring coating integrity and protection against physical damage and microbial contamination. The GRS/CS coatings with alternative molar proportions on mango surfaces significantly improved film adhesion and thickness compared to GRS alone. FTIR spectroscopy confirmed polymer film interactions with mango surfaces, highlighting its potential in food science and technology. Coating with GRS/CS blends effectively reduced post-harvest diseases, delayed ripening, and maintained mango quality during storage. Overall, GRS/CS blends offer a promising solution for extending mango shelf life, with potential for similar applications to other produce types.

## Figures and Tables

**Table 1:** Quantities of polymers and solvents for GRS/CS blends with varied molar ratios (total volume: 100 mL).

Sample Named	Ratio of GRS and Chitosan		Weight of GRS and Chitosan		Volume of Solvent	
	(%mol)		(g)		(mL)	
	GRS	Chitosan	GRS	Chitosan	H_2_O	CH_3_COOH
						(0.1 M)
GRS100/CS0	100	0	1.98	0.00	100	0
GRS70/CS30	70	30	1.39	0.60	70	30
GRS50/CS50	50	50	0.99	1.00	50	50
GRS30/CS70	30	70	0.60	1.40	30	70
GRS0/CS100	0	100	0.00	2.00	0	100

**Table 2:** Physical and chemical properties of GRS/CS blend solutions and films with varying molar compositions.

Properties		GRS100/CS0	GRS70/CS30	GRS50/CS50	GRS30/CS70	GRS0/CS100
Solution	pH	6.81 ± 0.03	5.63 ± 0.07	5.22 ± 0.11	4.55 ± 0.08	3.68 ± 0.04
	Viscosity (cP) at 29 ± 1 °C	27.3 ± 0.8	34.5 ± 0.5	66.5 ± 0.7	136.5 ± 0.4	667.5 ± 0.6
Film	Appearance of film	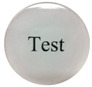	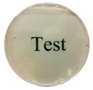	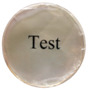	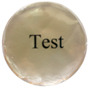	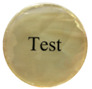
	Density (g/cm^3^)	1.88 ± 0.12	1.61 ± 0.08	1.53 ± 0.06	1.41 ± 0.03	1.09 ± 0.10
	Water solubility (%)	N/A	N/A	26.75 ± 0.50	19.94 ± 1.12	12.31 ± 0.59

## Data Availability

Data are contained within the article. The original contributions presented in the study are included in the article, further inquiries can be directed to the corresponding author.

## References

[1] Kumari C., Sharma M., Kumar V., Sharma R., Kumar V., Sharma P., Irfan M. (2022). Genome editing technology for genetic amelioration of fruits and vegetables for alleviating post-harvest loss. Bioengineering.

[2] Barbosa C.H., Andrade M.A., Vilarinho F.L., Fernando A.L., Silva A.S. (2021). Active edible packaging. Encyclopedia.

[3] Shahbandeh M. (2024). Global Mango Production 2000–2022. https://www.statista.com/statistics/577951/world-mango-production/.

[4] de Oliveira K.Á.R., da Conceição M.L., de Oliveira S.P.A., Lima M.D.S., de Sousa Galvão M., Madruga M.S., de Souza E.L. (2020). Postharvest quality improvements in mango cultivar Tommy Atkins by chitosan coating with *Mentha piperita* L. essential oil. J. Hortic. Sci. Biotechnol..

[5] Hoque M.I., Chowhan S., Kamruzzaman M. (2018). Physiological changes and shelf life of mango (*Mangifera indica* L.) Influenced by post harvest treatments. SAARC J. Agric..

[6] Busatto N., Vittani L., Farneti B., Khomenko I., Caffini M., Faccini S., Costa F. (2022). Physiological and molecular characterization of the late ripening stages in *Mangifera indica* cv Keitt. Postharvest Biol. Technol..

[7] Rastegar S., Hassanzadeh Khankahdani H., Rahimzadeh M. (2019). Effectiveness of alginate coating on antioxidant enzymes and biochemical changes during storage of mango fruit. J. Food Biochem..

[8] Ntsoane M.L., Zude-Sasse M., Mahajan P., Sivakumar D. (2019). Quality assesment and postharvest technology of mango: A review of its current status and future perspectives. Sci. Hortic..

[9] Díaz-Montes E., Castro-Muñoz R. (2021). Edible films and coatings as food-quality preservers: An overview. Foods.

[10] Berihu H., Zegeye A. (2022). Enhancement of quality and storability of Avocado (*Persea americana*) fruit using a blend of aloe vera gel and corn starch as surface coating. Int. J. Food Eng. Technol..

[11] Oyom W., Xu H., Liu Z., Long H., Li Y., Zhang Z., Bi Y., Tahergorabi R., Prusky D. (2022). Effects of modified sweet potato starchedible coating incorporated with cumin essential oil on storage quality of ‘early crisp’. LWT.

[12] Ghoshal G., Chopra H. (2022). Impact of apricot oil incorporation in tamarind starch/gelatin based edible coating on shelf life of grape fruit. J. Food Meas. Charact..

[13] Rather J.A., Makroo H.A., Showkat Q.A., Majid D., Dar B.N. (2022). Recovery of gelatin from poultry waste: Characteristics of the gelatin and lotus starch-based coating material and its application in shelf-life enhancement of fresh cherry tomato. Food Packag. Shelf Life.

[14] Cazón P., Velazquez G., Ramírez J.A., Vázquez M. (2017). Polysaccharide-based films and coatings for food packaging: A review. Food Hydrocoll..

[15] Oladzadabbasabadi N., Nafchi A.M., Ariffin F., Wijekoon M.J.O., Al-Hassan A.A., Dheyab M.A., Ghasemlou M. (2022). Recent advances in extraction, modification, and application of chitosan in packaging industry. Carbohydr. Polym..

[16] Yan D., Li Y., Liu Y., Li N., Zhang X., Yan C. (2021). Antimicrobial properties of chitosan and chitosan derivatives in the treatment of enteric infections. Molecules.

[17] Román-Doval R., Torres-Arellanes S.P., Tenorio-Barajas A.Y., Gómez-Sánchez A., Valencia-Lazcano A.A. (2023). Chitosan: Properties and its application in agriculture in context of molecular weight. Polymers.

[18] Hesami A., Kavoosi S., Khademi R., Sarikhani S. (2021). Effect of chitosan coating and storage temperature on shelf-life and fruit quality of Ziziphus mauritiana. Int. J. Fruit Sci..

[19] Saki M., ValizadehKaji B., Abbasifar A., Shahrjerdi I. (2019). Effect of chitosan coating combined with thymol essential oil on physicochemical and qualitative properties of fresh fig (*Ficus carica* L.) fruit during cold storage. J. Food Meas. Charact..

[20] Parvin N., Rahman A., Roy J., Rashid M.H., Paul N.C., Mahamud M.A., Kader M.A. (2023). Chitosan coating improves postharvest shelf-life of mango (*Mangifera indica* L.). Horticulturae.

[21] Ojeda G.A., Arias Gorman A.M., Sgroppo S.C., Zaritzky N.E. (2021). Application of composite cassava starch/chitosan edible coating to extend the shelf life of black mulberries. J. Food Process. Preserv..

[22] Shapi’i R.A., Othman S.H., Nordin N., Basha R.K., Naim M.N. (2020). Antimicrobial properties of starch films incorporated with chitosan nanoparticles: In vitro and in vivo evaluation. Carbohydr. Polym..

[23] da Costa J.C.M., Miki K.S.L., da Silva Ramos A., Teixeira-Costa B.E. (2020). Development of biodegradable films based on purple yam starch/chitosan for food application. Heliyon.

[24] Lee H.M., Kim M.H., Yoon Y.I., Park W.H. (2017). Fluorescent property of chitosan oligomer and its application as a metal ion sensor. Mar. Drugs.

[25] (2012). Standard Test Method for Tensile Properties of Thin Plastic Sheeting.

[26] Wongkaew M., Sangta J., Chansakaow S., Jantanasakulwong K., Rachtanapun P., Sommano S.R. (2021). Volatile profiles from over-ripe purée of Thai mango varieties and their physiochemical properties during heat processing. PLoS ONE.

[27] Kim K.M., Son J.H., Kim S.K., Weller C.L., Hanna M.A. (2006). Properties of Chitosan Films as a Function of pH and Solvent Type. J. Food Sci. E Food Eng. Phys. Prop..

[28] Xu J., Liu K., Chang W., Chiou B.S., Chen M., Liu F. (2022). Regulating the Physicochemical Properties of Chitosan Films through Concentration and Neutralization. Foods.

[29] Abidin M.Z.A.Z., Julkapli N.M., Juahir H., Azaman F., Abidin I.Z., Sulaiman N.H. (2015). Fabrication and properties of chitosan with starch for packaging application. Malays. J. Anal. Sci..

[30] Juliano B.O. (1979). The chemical basis of rice grain quality. Proceedings of the Workshop on Chemical Aspects of Rice Grain Quality.

[31] Pfister B., Zeeman S.C., Rugen M.D., Field R.A., Ebenhöh O., Raquin A. (2020). Theoretical and experimental approaches to understand the biosynthesis of starch granules in a physiological context. Photosynth. Res..

[32] Mathew S., Brahmakumar M., Abraham T.E. (2006). Microstructural imaging and characterization of the mechanical, chemical, thermal, and swelling properties of starch–chitosan blend films. Biopolymers.

[33] Seo S. (2006). Depolymerization and Decolorization of Chitosan by Ozone Treatment. Master’s Thesis.

[34] Bilbao-Sainz C., Sen Chiou B., Williams T., Wood D., Du W.X., Sedej I., Ban Z., Rodov V., Poverenov E., Vinokur Y. (2017). Vitamin D-fortified chitosan films from mushroom waste. Carbohydr. Polym..

[35] Ibrahim M.I.J., Sapuan S.M., Zainudin E.S., Zuhri M.Y.M. (2019). Physical, thermal, morphological, and tensile properties of cornstarch-based films as affected by different plasticizers. Int. J. Food Prop..

[36] Sharma M., Kulshrestha S. (2015). Colletotrichum gloeosporioides: An anthracnose causing pathogen of fruits and vegetables. Biosci. Biotechnol. Res. Asia.

[37] Le T.D., Viet Nguyen T., Muoi N.V., Toan H.T., Lan N.M., Pham T.N. (2022). Supply chain management of Mango (*Mangifera indica* L.) fruit: A review with a focus on product quality during postharvest. Front. Sustain. Food Syst..

[38] Marques K.M., Galati V.C., Fernandes J.D.R., Guimarães J.E.R., Silva J.P., Mattiuz B.H., Mattiuz C.F.M. (2016). Use of chitosan for the control of postharvest anthracnose and quality in avocados. Acta Hortic..

[39] Edirisinghe M., Ali A., Maqbool M., Alderson P.G. (2014). Chitosan controls postharvest anthracnose in bell pepper by activating defense-related enzymes. J. Food Sci. Technol..

[40] Ayón Reyna L.E., Uriarte Gastelum Y.G., Camacho Díaz B.H., Tapia Maruri D., López López M.E., López Velázquez J.G., Vega Garcia M.O. (2022). Antifungal activity of a chitosan and mint essential oil coating on the development of Colletotrichum gloeosporioides in papaya using macroscopic and microscopic analysis. Food Bioprocess Technol..

[41] Limon T., Birke A., Monribot-Villanueva J.L., Guerrero-Analco J.A., Altúzar-Molina A., Carrión G., Aluja M. (2021). Chitosan coatings reduce fruit fly (*Anastrepha obliqua*) infestation and development of the fungus Colletotrichum gloeosporioides in Manila mangoes. J. Sci. Food Agric..

[42] Qin Y., Li P., Guo Z. (2020). Cationic chitosan derivatives as potential antifungals: A review of structural optimization and applications. Carbohydr. Polym..

[43] Lopez-Moya F., Suarez-Fernandez M., Lopez-Llorca L.V. (2019). Molecular mechanisms of chitosan interactions with fungi and plants. Int. J. Mol. Sci..

[44] Goehring L. (2009). Drying and cracking mechanisms in a starch slurry. Phys. Rev. E.

[45] Izawa H., Ishisaka S., Saimoto H., Ifuku S. (2022). Drying-Induced Surface Wrinkles Generated on Chitosan Films Having Polyion Complex Skin Layers: Effects of Physical Properties of Skin Layers and Substrates on Surface Wrinkling upon Drying. Bull. Chem. Soc. Jpn..

[46] Morawetz H. (1999). On the versatility of fluorescence techniques in polymer research. J. Polym. Sci. Part A Polym. Chem..

[47] Roman M., Nechita P., Vasile M.-A., Cantaragiu Ceoromila A.-M. (2023). Barrier and Antimicrobial Properties of Coatings Based on Xylan Derivatives and Chitosan for Food Packaging Papers. Coatings.

[48] Wongs-Aree C., Nguyen H.T., Noichinda S. (2023). Improved Postharvest Techniques for Fruit Coatings. New Advances in Postharvest Technology.

[49] Bertuzzi M.A., Castro Vidaurre E.F., Armada M., Gottifredi J.C. (2007). Water vapor permeability of edible starch based films. J. Food Eng..

[50] Mangaraj S., Goswami T.K., Panda D.K. (2015). Modeling of gas transmission properties of polymeric films used for MA packaging of fruits. J. Food Sci. Technol..

[51] Luchese C.L., Pavoni J.M.F., Dos Santos N.Z., Quines L.K., Pollo L.D., Spada J.C., Tessaro I.C. (2018). Effect of chitosan addition on the properties of films prepared with corn and cassava starches. J. Food Sci. Technol..

[52] Fadiji T., Rashvand M., Daramola M.O., Iwarere S.A. (2023). A Review on Antimicrobial Packaging for Extending the Shelf Life of Food. Processes.

[53] Mitchell J.R., MacNaughtan W., Foster T.J., Harabagiu V., Song Y., Zheng Q. (2010). Comparison of the Mechanical Properties of Cellulose and Starch Films. Biomacromolecules.

[54] Bertoft E. (2017). Understanding Starch Structure: Recent Progress. Agronomy.

[55] D’Angelo G., Elhussieny A., Faisal M., Fahim I.S., Everitt N.M. (2018). Mechanical Behavior Optimization of Chitosan Extracted from Shrimp Shells as a Sustainable Material for Shopping Bags. J. Funct. Biomater..

[56] Prasad K., Sharma R.R., Srivastav M. (2016). Postharvest treatment of antioxidant reduces lenticel browning and improves cosmetic appeal of mango (*Mangifera indica* L.) fruits without impairing quality. J. Food Sci. Technol..

[57] Bansal H., Singh S., Sharma A., Sundaramurthy S., Mehta S.K., Pandey M., Deshmukh K. (2024). Polymer nanocomposite films and coatings for antimicrobial and antifungal applications. Polymer Nanocomposite Films and Coatings.

[58] Heredia-Guerrero J.A., Benítez J.J., Domínguez E., Bayer I.S., Cingolani R., Athanassiou A., Heredia A. (2014). Infrared and Raman spectroscopic features of plant cuticles: A review. Front. Plant Sci..

[59] Prinsloo L.C., du Plooy W., van der Merwe C. (2004). Raman spectroscopic study of the epicuticular wax layer of mature mango (*Mangifera indica*) fruit. J. Raman Spectrosc..

[60] Dubis E.N., Dubis A.T., Morzycki J.W. (1999). Comparative analysis of plant cuticular waxes using HATR FT-IR reflection technique. J. Mol. Struct..

[61] Kizil R., Irudayaraj J., Seetharaman K. (2002). Characterization of irradiated starches by using FT-Raman and FTIR spectroscopy. J. Agric. Food Chem..

[62] Soe M.T., Pongjanyakul T., Limpongsa E., Jaipakdee N. (2021). Films Fabricated with Native and Ball-Milled Modified Glutinous Rice Starch: Physicochemical and Mucoadhesive Properties. Starch.

[63] Wang S., Wang J., Zhang W., Li C., Yu J., Wang S. (2015). Molecular order and functional properties of starches from three waxy wheat varieties grown in China. Food Chem..

[64] Xu Y.X., Kim K.M., Hanna M.A., Nag D. (2005). Chitosan–starch composite film: Preparation and characterization. Ind. Crops Prod..

[65] Rakkapao N., Vao-Soongnern V., Masubuchi Y., Watanabe H. (2011). Miscibility of chitosan/poly (ethylene oxide) blends and effect of doping alkali and alkali earth metal ions on chitosan/PEO interaction. Polymers.

[66] Gieroba B., Sroka-Bartnicka A., Kazimierczak P., Kalisz G., Lewalska-Graczyk A., Vivcharenko V., Przekora A. (2022). Surface chemical and morphological analysis of chitosan/1, 3-β-d-glucan polysaccharide films cross-linked at 90 °C. Int. J. Mol. Sci..

[67] Queiroz M.F., Melo K.R.T., Sabry D.A., Sassaki G.L., Rocha H.A.O. (2014). Does the use of chitosan contribute to oxalate kidney stone formation?. Mar. Drugs.

[68] Hadthamard N., Chaumpluk P., Buanong M., Boonyaritthongchai P., Wongs-Aree C. (2019). Effects of Multilayer Coating of Chitosan and Polystyrene Sulfonate on Quality of ‘Nam Dok Mai No. 4’ Mango. Int. J. Agric. Biosyst. Eng..

[69] Felicia W.X.L., Rovina K., Nur’Aqilah M.N., Vonnie J.M., Erna K.H., Misson M., Halid N.F.A. (2022). Recent advancements of polysaccharides to enhance quality and delay ripening of fresh produce: A review. Polymers.

[70] Kumari M., Mahajan H., Joshi R., Gupta M. (2017). Development and structural characterization of edible films for improving fruit quality. Food Packag. Shelf-Life.

[71] Jongsri P., Wangsomboondee T., Rojsitthisak P., Seraypheap K. (2016). Effect of molecular weights of chitosan coating on postharvest quality and physicochemical characteristics of mango fruit. LWT-Food Sci. Technol..

[72] Wongkhot A., Rattanapanone N., Chanasut U. (2012). BrimA, total acidity and total soluble solids correlate to total carotenoid content as indicators of the ripening process of six thai mango fruit cultivars. Chiang Mai Univ. J. Nat. Sci..

[73] Silva G.M.C., Silva W.B., Medeiros D.B., Salvador A.R., Cordeiro M.H.M., da Silva N.M., Santana D.B., Mizobutsi G.P. (2017). The chitosan affects severely the carbon metabolism in mango (*Mangifera indica* L. cv. Palmer) fruit during storage. Food Chem..

